# A DNA telomerase vaccine for canine cancer immunotherapy

**DOI:** 10.18632/oncotarget.26927

**Published:** 2019-05-21

**Authors:** Jessie Thalmensi, Elodie Pliquet, Christelle Liard, Gabriel Chamel, Christine Kreuz, Thomas Bestetti, Marie Escande, Anna Kostrzak, Anne-Sophie Pailhes-Jimenez, Emmanuèle Bourges, Marion Julithe, Ludovic Bourre, Olivier Keravel, Pascal Clayette, Thierry Huet, Simon Wain-Hobson, Pierre Langlade-Demoyen

**Affiliations:** ^1^ Invectys, Paris BioPark, Paris 75013, France; ^2^ ImmunoPharmacology and Biosafety Lab, Bertin Pharma/CEA, Fontenay-aux-Roses 92265, France; ^3^ Eiffelvet, Paris 75015, France; ^4^ Molecular Retrovirology Unit, Institut Pasteur, CNRS-URA 3015, Paris 75015, France

**Keywords:** DNA vaccine, canine TERT, cancer, immunotherapy, Electro-Gene-Transfer

## Abstract

Telomerase reverse transcriptase (TERT) is highly expressed in more than 90% of canine cancer cells and low to absent in normal cells. Given that immune tolerance to telomerase is easily broken both naturally and experimentally, telomerase is an attractive tumor associated antigen for cancer immunotherapy. Indeed, therapeutic trials using human telomerase peptides have been performed. We have developed an immunogenic yet catalytically inactive human telomerase DNA construct that is in clinical trials with patients presenting solid tumors. Paralleling this human construct, we have developed a canine telomerase DNA vaccine, called pDUV5. When administered intradermally to mice combined with electrogene transfer, pDUV5 induced canine TERT specific cytotoxic T-cells as measured by IFN-γ ELISpot assay. Intradermal vaccination of healthy dogs with 400 μg of pDUV5 generated strong, broad and long lasting TERT specific cellular immune responses. *In vitro* immunization with cTERT peptides revealed the maintenance of cTERT specific T-cells in PBMCs from tumor bearing dogs showing that this repertoire was not depleted. This study highlights the potential of pDUV5 as a cancer vaccine and supports its evaluation for the treatment of spontaneous canine tumors.

## INTRODUCTION

Like humans, dogs have seen increased life expectancy and with it a rising cancer burden. Canine cancers occur with a similar incidence to that of humans, the difference being that the treatment panel is grossly limited. New therapeutic veterinary approaches are greatly needed. One of the few novel products to come onto the pet market is DNA vaccination targeting the tumor associated antigen (TAA) canine tyrosinase. Xenogeneic DNA vaccines were designed to bypass central immune tolerance. Indeed, recent studies reported the safety and efficacy of a DNA vaccine encoding murine tyrosinase for malignant melanoma of the digit of dogs [1] and ONCEPT, a DNA vaccine encoding human tyrosinase for oral malignant melanoma in dogs [2] was approved by the U.S. Department of Agriculture in 2010.

Therapeutic vaccination represents a potential strategy since it induces memory immune responses and can easily be associated with conventional treatments. Particularly for pet cancers treatment costs are a limitation meaning that any TAA should be expressed in large numbers of diverse cancers [3]. Telomerase reverse transcriptase (TERT) is a very attractive target for cancer immunotherapy. TERT is the rate-limiting catalytic subunit of the telomerase complex which synthetizes telomeric DNA at chromosome ends [4]. It prevents apoptosis through the telomere dependent pathway so promoting cell immortality. Indeed, TERT driven immortalization is one of the hallmarks of oncogenesis [5]. Human TERT is overexpressed in >90% human cancers meaning that is it close to being a universal TAA. Tolerance to telomerase can readily be broken both naturally and experimentally [6]. We have developed a powerful hTERT recombinant DNA plasmid (INVAC-1) that elicited cell mediated memory responses and curtailed tumor growth in mice yet was devoid of any immortalizing activity [7]. Another group has demonstrated similar findings using a secreted hTERT construct [8]. Both studies have led to ongoing phase I clinical trials (NCT02301754 and NCT02327468).

TERT is also overexpressed in the majority (>90%) of canine cancer cells regardless of their origin [9] and there is a good correlation between TERT expression and telomerase activity in dog tissues [10]. Accordingly like its human counterpart, cTERT is considered to be a near universal tumor associated antigen (TAA) [11] justifying its use in clinical immunotherapy as a treatment for dog cancers [9].

DNA vaccines offer the flexibility to incorporate easily multiple genes for tumor antigens and/or immunostimulatory molecules [12, 13]. Improvements in Electro-Gene-Transfer (EGT, also known as DNA electroporation), notably the use of a sequence of high volt-low volt pulses, have greatly increased the efficiency of the DNA vaccination [14]. Today, many ongoing clinical trials employ DNA vaccination against infectious diseases or cancer [15]. DNA has the advantage that manufacturing is derisked, relatively inexpensive and has a shelf life measured in years. In this context, we developed pDUV5, a DNA plasmid encoding the canine TERT equivalent to INVAC-1 as an immunotherapeutic agent to fight dog cancers. When administered using EGT it induced strong cytotoxic CD8 T-cell responses in mice and cTERT specific immune responses in healthy beagle dogs. Furthermore, we showed the maintenance of a naturally occurring repertoire of cTERT specific T-cells in tumors bearing dogs. These pharmacological data support the use of pDUV5 in clinical trials for the immunotherapy of a broad range of canine tumors.

## RESULTS

### pDUV5 design and characterization

**Figure 1:**
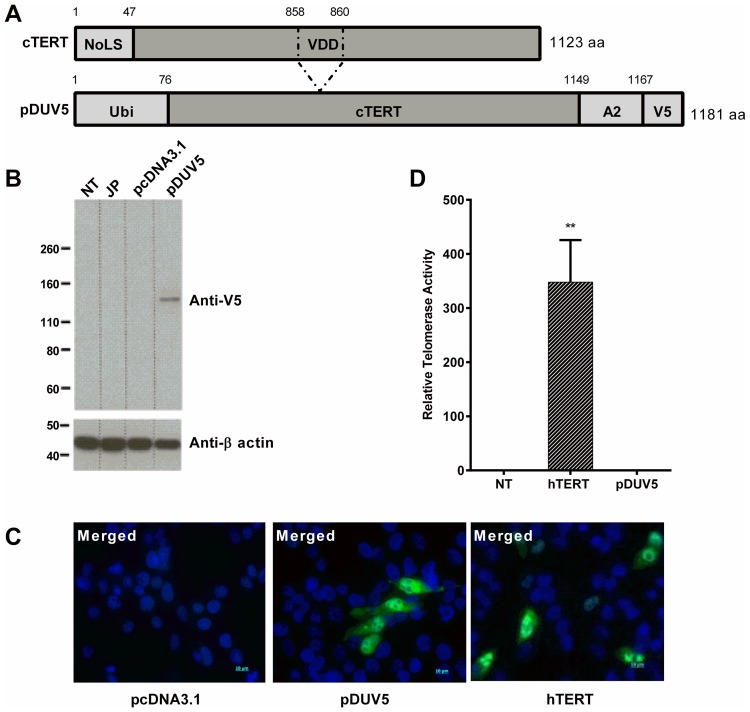
*In vitro* characterization of pDUV5 protein. (**A**) Schematic maps and alignments of wild type cTERT and pDUV5 proteins. VDD: deletion in catalytic site at amino acid positions 858-860. NoLS, nucleolar localization signal; Ubi, ubiquitin; Flu, influenza A HLA-A2 restricted epitope; V5, V5 tag. (**B**) Expression of pDUV5 protein monitored 24 h post-transfection in HEK293T cells. Protein was detected using an anti-V5 mouse monoclonal antibody. pcDNA3.1 empty vector backbone as negative control. JP: jetPRIME. NT: non-treated cells. β-actin protein detection was used as a loading control assessment. (**C**) Intracellular localization of wild type hTERT and pDUV5 proteins in transfected QT6 cells visualized 24 h post-transfection with a rabbit anti-hTERT antibody or a mouse anti-V5 antibody respectively and a goat anti-rabbit-Alexa Fluor 488 conjugate or a goat anti-mouse antibody-Alexa Fluor 488 conjugate (green fluorescence) respectively. pcDNA3.1 vector as negative control. The nuclei were stained with DAPI (blue). The cells were analyzed for both fluorescence wavelengths (merged) upon fluorescence microscopy. (**D**) Neutralization of pDUV5 telomerase catalytic activity. Total cell proteins were extracted from wild type hTERT and pDUV5 transfected CRFK cells and telomerase activity was assessed by Telomeric Repeat Amplification Protocol (TRAP) assay. Relative Telomerase Activity (RTA; sample/positive control ratio) of pDUV5 compared to wild type hTERT and non-treated (NT) CRFK cells are displayed (n = 3 for 2.1 μg of total protein samples) using absorbance measurements values (OD450/690 nm). Mann-Whitney non-parametric test against non-treated CRFK cells, ^**^p < 0.01**.**

We generated a cTERT construct based on the same principles as the INVAC-1 telomerase plasmid in human phase I trials (NCT02301754 [7]). Briefly, the construct carries two safety features, notably deletion of the nucleolar localization sequence (NoLS) and deletion of the catalytic VDD triplet leading to an inactive form of the enzyme. To facilitate processing by the proteasome, cTERT was fused to ubiquitin following the so-called N-end rule (destabilizing residue at N-terminal position) which orients degradation through the ubiquitin-dependent proteasome pathway and subsequently for antigen presentation by the MHC class I [16]. At flu epitope and the V5 tag were added to the C-terminus allowing the expressed fusion protein to be characterized, there being no cTERT specific antibodies available. The pcDNA3.1 based plasmid is referred to as pDUV5 (Figure 1A).

Canine TERT-V5 protein expression was assessed by Western blotting 24 hours after pDUV5 transfection of HEK293T cells. As can be seen from Figure 1B a single band was identified which corresponds well with the expected molecular weight of 130 kDa. When analyzed by immunofluorescence, overexpressed recombinant cTERT was mainly localized in the cytoplasm and nucleus but not in the nucleolus, which contrasts with normal human TERT (Figure 1C) that is found there in abundance in the nucleolus [17].

To confirm inactivation of cTERT, telomerase activity was determined in transfected CRFK cells using the telomeric repeat amplification assay. Relative telomerase activity (RTA) data derived using pDUV5 transfected cell extracts showed that cTERT was completely devoid of telomerase activity (Figure 1D). As a positive control, the cell lysates from CRFK cells transfected with wild type human TERT (hTERT) were used. These results demonstrate that pDUV5 encoded cTERT fusion protein displayed the characteristics and properties expected of its design and a prerequisite for vaccination.

### pDUV5 induces strong cytotoxic CD8 T-cell responses

For immunization the pDUV5 insert was subcloned into the NTC8685-eRNA41H-BamHI-XbaI expression vector and named pNTC-DUV5. The vector encodes a small RNA transcript that folds up into a hairpin so enhancing cellular immune responses approximately 2 fold [18]. Importantly does not encode an antibiotic resistance gene [19, 20] so making it environmentally friendly.

**Figure 2:**
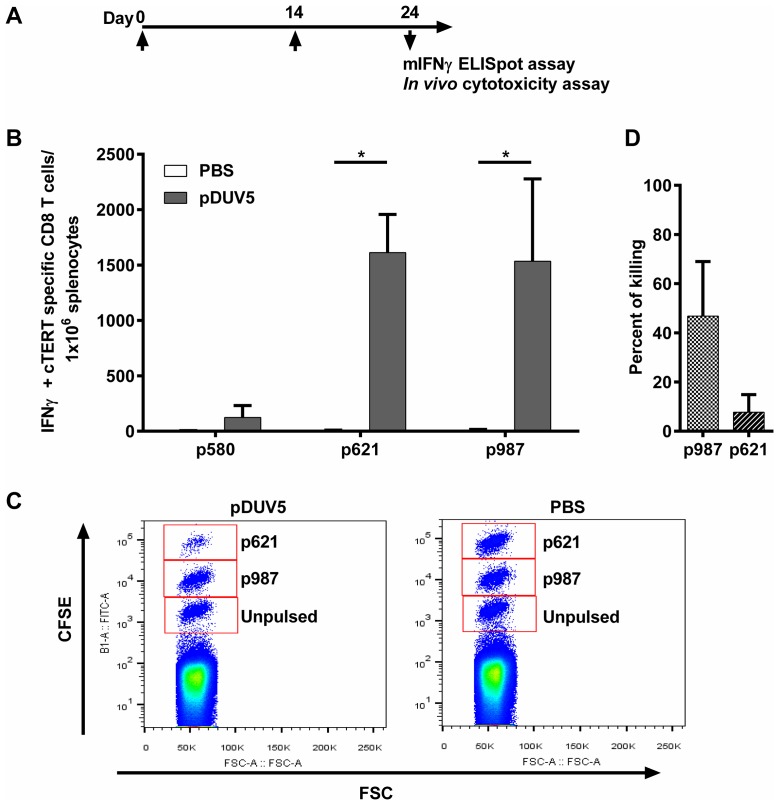
pDUV5 induces cTERT cytotoxic specific T-cell responses in mice. (**A**) C57BL/6 mice (4 mice per group) were immunized twice at D0 and D14. Ten days later, an IFN-γ ELISpot assay or an *in vivo* cytotoxicity assay were performed. (**B**) Splenocytes from immunized mice were stimulated with H2-Db restricted cTERT peptides. IFN-γ cTERT specific CD8 T-cells/10^6^ splenocytes are represented as means ± SD. Mann-Whitney non-parametric test against mice control immunized with PBS, ^*^p < 0.05. (**C**) C57BL/6 mice (10 mice per group) were immunized twice (D0 and D14). At D24, syngeneic splenocytes pulsed with individual cTERT peptides restricted to H2-Db (either p621 or p987) or unpulsed were labeled with CFSE and injected IV to immunized mice. After 15 hours, the disappearance of peptide pulsed cells in spleens was analyzed by flow cytometry. (**D**) Percent killing was presented as means ± SD.

The plasmid pDUV5 was first tested in mice, the vaccination protocol being summarized in Figure 2A. As a prerequisite for detecting cellular immune responses, H2-Db restricted cTERT peptides were predicted *in silico* and synthesized (Table 1). As judged by an ELISpot IFN-γ assay ten days after the second immunization, pDUV5 immunized C57BL/6 mice generated significant cTERT specific CD8 T-cell responses compared to control mice immunized with PBS for 2 of 3 peptides (p621 and p987; *p* < 0.05) (Figure 2B). *In vivo* CD8 T-cells cytotoxic activity was assessed by flow cytometry using CFSE-labeled and peptide pulsed splenocytes in mice. Using the same immunization protocol there was a decrease of highly-labeled CFSE cells pulsed with peptide p621 and a slight decrease of medium-labeled CFSE cells pulsed with peptide p987 (Figure 2C). Approximately 30% of p987 pulsed cells and 62% of p621 pulsed-cells were killed in pDUV5 immunized mice (Figure 2D). No toxicity was observed in terms of body weight, morbidity or mortality. Taken together, these results confirm that the pDUV5 construct is able to induce cTERT cytotoxic specific immune responses *in vivo*.

**Table 1:** cTERT restricted peptides predicted by *in silico* algorithms

Peptide	Sequence	MHC	Numbering	Mouse strain
p580	RQLFNSVHL	H2-Db	cTERT	C57BL/6
p621	RPIVNMDYI			
p987	TVYMNVYKI			

### *In vivo* cTERT specific cellular immune responses

**Figure 3:**
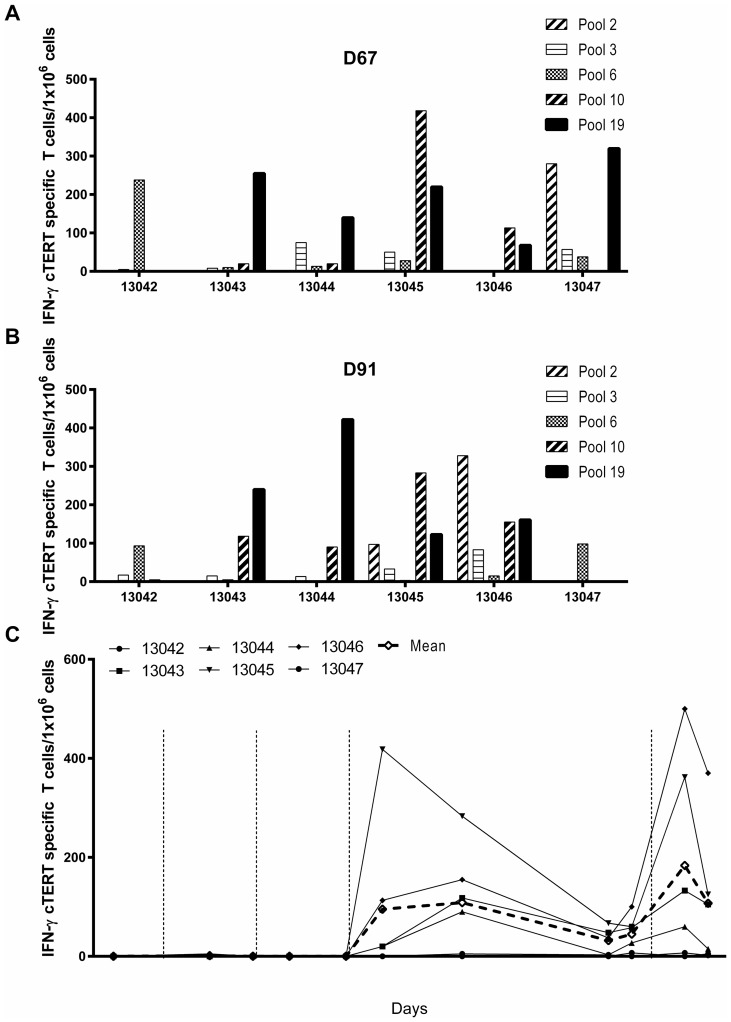
Immunogenicity of pNTC-DUV5 in beagle dogs. (**A**) Six naïve healthy beagle dogs were immunized intradermally with 400 μg of pNTC-DUV5 at days 0, 29, 57 and 148. (**B**) PBMCs from treated dogs were analyzed in an ELISpot IFN-γ assay at D67 and D91 using 19 peptides pools overlapping the cTERT proteins. Higher IFN-γ cTERT specific T-cells/10^6^ PBMCs were obtained with five peptides pools (pools 2, 3, 6, 10 and 19). (**C**) For the kinetic of the cTERT specific T-cell response, blood was collected before the first immunization at D-14 then at regular time points up to D165. PBMCs were purified by Ficoll separation, and the response was measured by ELISpot IFN-γ after stimulation by the peptides pool 10. IFN-γ cTERT specific T-cells/10^6^ PBMCs are represented over the times, filled symbols indicate individual animals whereas open diamonds correspond to the group average.

Six naïve beagle dogs were injected intradermally at days 1, 29, 57 and 148 with 400 μg of pNTC-DUV5 followed by EGT. Canine TERT specific T-cell responses were monitored in PBMCs from D-14 to D165 with an IFN-γ ELISpot assay using 19 pools of overlapping 15-mer peptides, each pool covering ~6% of cTERT. At D67 and D91, strong responses were observed for five pools (pools 2, 3, 6, 10 and 19; Table 2) and are illustrated in Figure 3A and 3B. A broad repertoire of T-cells against numerous cTERT peptides was induced in all dogs, although with considerable heterogeneity. Longitudinal T-cell responses in PBMCs are shown for pool 10 peptides (Figure 3C). After the third immunization significant cTERT specific T-cell responses were observed at D91 (mean # spots: 109, *p* = 0.015) compared to baseline (D-14). The immune response was long lasting since it was still detected at D142 (mean # spots: 45, *p* = 0.0022) i.e. 85 days after the third immunization. Ten days after the fourth and final immunization (D158) a higher and faster immune responses were observed (mean # spots: 184, *p*=0.015) indicating establishment of a cTERT specific memory response. No toxicity was observed and no changes in body weight, hematology, coagulation and blood chemistry parameters were noted when compared to pre-dose values.

**Table 2:** cTERT peptide library

Pool	cTERT sequence covered by pool	Residues	
Pool A	AKLSLQELTWKMKVRDCTWLHGNPGACCVPAAEHRRREEILARFLVLVDGHIYVVKLLRSFFYVTETTFQKNRLFFYRKSVW	490 to 580	*In vitro* immunization
Pool 2	CVPWGARPPPAAPCFRQVSCLKELVARVVQRLCERGARNVLAFGFALLDGARGGPPVAFTTSVRSYL	57 to 123	*In vivo* dog studies
Pool 3	VAFTTSVRSYLPNTVTETLRGSGAWGLLLRRVGDDVLTHLLARCALYLLVAPSCAYQVCGPPLYDLC	113 to 179	
Pool 6	EGGPPGTRPTTPAWHPYPGP QGVPHDPAHP ETKRFLYCSG GRERLRPSFLLSALPPTLSGARKLVET	281 to 347	
Pool 10	DCTWLHGNPGACCVPAAEHRRREEILARFLVLVDGHIYVVKLLRSFFYVTETTFQKNRLFFYRKSVW	505 to 571	
Pool 19	QLPFNQPVRKNPSFFLRVIADTASCCYSLLKARNAGLSLGAKGASGLFPSEAARWLCLHAFLLKLAH	1009 to 1075	

### Natural existing canine TERT specific T-cell repertoire

**Figure 4:**
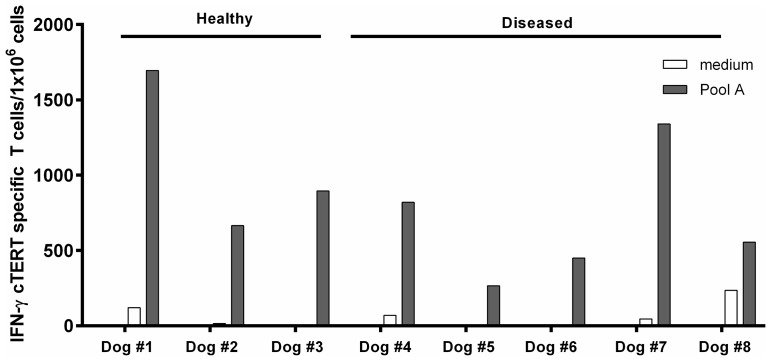
*In vitro* immunization assays with dogs PBMCs. Frozen PBMCs from healthy and tumor bearing dogs were incubated during 24 hours with cGM-CSF and IL-4. After 15 days, culture cells were recovered and analyzed by an IFN-γ ELISpot assay after stimulation with the peptides of pool A. Results are expressed as IFN-specific T-cells/10^6^ PBMCs.

The prerequisite for a successful antitumor syngeneic DNA vaccination is that immunological self-tolerance to tumor antigens be overcome. This is especially important in tumor bearing dogs where somatic changes in tumor cell DNA can result in manipulation of immune responses. In view of this we investigated the existence of cTERT specific T-cell repertoire in tumor bearing dogs along with healthy dogs as controls. Adapting a protocol designed for human PBMCs [21], we performed *in vitro* immunizations of PBMCs from three healthy and five diseased dogs (Table 3). Fifteen days after stimulation, T-cell responses to a pool of 18 overlapping 15-mer cTERT peptides representing merely 8% of cTERT (Pool A, Table 2) were detected for all animals confirming the existence of a naturally occurring repertoire of cTERT specific T-cell either in healthy or diseased dogs (Figure 4).

**Table 3:** Data for healthy and tumor bearing dogs

ID	Breed	Age (years)	Sex	Pathology
Dog #1	Boxer	7	F	Healthy
Dog #2	Jack Russel	5	F	Healthy
Dog #3	Rottweiler	9	F	Healthy
Dog #4	Labrador	8	F	Mastocytoma grade II
Dog #5	Labrador	12	M	Tumor hypothesis (liver/right adrenal)
Dog #6	Bernese Mountain Dog	9	M	Neoplasm + lung metastasis
Dog #7	Cavalier King Charles	10	F	Bone tumor
Dog #8	Shetland Sheepdog	2.5	M	Histiocytoma

To show that pNTC-DUV5 can induce specific cTERT T cell responses in animals with neoplasias, five pet dogs with neoplasias and three pet dogs as controls were used.

## DISCUSSION

Paralleling its human counterpart currently in clinical trials (NCT02301754), a ubiquitin-canine TERT DNA construct was synthesized given that ubiquitin-fused DNA vaccines have showed a significant improvement of the antigen specific cellular immune response [22]. Although tagged by ubiquitin, overexpression was so strong that some recombinant cTERT made its way to the nucleus. However, it was excluded from the nucleolus unlike wild type TERT, which is most abundant in this compartment [17]. As the TRAP assay showed the fusion protein to be totally devoid of enzymatic activity, in keeping with the role of the VDD amino acid triplet in telomerase function [23], there is no danger of recombinant telomerase immortalizing target cells.

DNA vaccination *per se* alone is known to be poorly immunogenic requiring electrogene transfer to greatly increase DNA uptake. This combination has demonstrated its capacity to induce a robust and long lasting antigen-specific T-cell response in mice [14], dogs [24] or humans [25]. EGT also acts as an adjuvant by improving transfection of cells, increasing antigen expression and by generating local inflammation leading to recruitment of APCs [26, 27]. In mice, a specific cellular immune response was detected after two immunizations with 100 μg of pDUV5, showing that our vaccine is immunogenic *in vivo* and that the cTERT specific CD8 T-cells induced can recognize and lyse target cells showing that they are functional CTLs which are known to play an important role in anti-tumor immunity.

pDUV5 was administered intradermally, the skin being an attractive site for vaccination given the abundance of leukocytes compared for example to muscle [28]. EGT leads to the transfection of APCs that migrate to the lymph nodes allowing the priming and differentiating of CD8 T-cells into cytolytic effectors [29]. In healthy dogs, electroporated pNTC-DUV5 induced a cTERT specific cellular immune response after three immunizations. Strongest responses were observed at D91, i.e. 34 days after the third immunization. Other DNA vaccine studies in dogs confirm that 3 immunizations are also needed to induce specific cellular immune responses [24, 30, 31]. The broad response induced by pNTC-DUV5 against numerous cTERT epitopes spanning the entire protein confirmed the advantage of using full-length protein as compared to individual epitopes used in peptide vaccine development. Moreover, by encoding full-length cTERT, the whole MHC diversity among the dog population is expected to be covered [32]. Eighty-five days (D142) after the third immunization, cTERT specific T-cells were still detected (Figure 3). A fourth immunization (D148) increased the specific immune response in as little as 10 days as expected from a memory response. The findings confirm that multiple doses of the vaccine can be administered resulting in enhanced specific immune responses [33]. These results indicate induction of a long lasting memory response which has the ability to expand rapidly upon encounter with the same antigen a second-time round and thereby protecting patients from relapses [34].

Numerous studies have also shown that T-cell precursors against overexpressed TAAs can be found both in cancer patients and healthy individuals [6, 35–37]. It turns out that experimental breaking of tolerance to telomerase is surprisingly easy. While cells bearing high-affinity receptors are no doubt depleted, those with intermediate or low-affinity main remain [38]. cTERT specific T-cells could be readily identified in peripheral canine PBMCs and was also true for dogs with tumors. Just as for humans and mice, a naturally occurring telomerase specific T-cell repertoire exists in both healthy and, more importantly, tumor bearing dogs, showing that this repertoire is not altered, depleted, or suppressed by disease. As MHC diversity is much less than for humans the immune responses identified here will reflect those of a much larger population.

A previous study used a secreted form of cTERT fused to the *E. coli* heat labile endotoxin. Vaccination was via DNA electroporation followed by adenovirus boosting [31]. Via the intradermal route 3 × 400 μg of pDUV5 has been proved to be as immunogenic as 5 × 2.5 mg of their product V1J-dTERT.LTB_opt_ construct via the intramuscular route. There are too many differences between the two constructs, electrogene transfer protocols and ELISpot peptides targets to be able to understand these differences, although our electroporation protocol was first optimized using luciferase constructs in mice [14]. A subsequent report involving adenovirus-cTERT priming followed by 5–13 × 5 mg cTERT/EGT boosts resulted in increased overall survival [39]. For a viable canine vaccine cost is a major limitation, far more so than for humans. Given the strong immune responses generated by pDUV5 with ≤10–50 fold less DNA without any adenovirus prime or boost, pDUV5 may well be a realistic option for treating canine tumors. As the immunogenicity and safety of pDUV5 is demonstrated in dogs and mice, the next step is clinical evaluation of efficacy in spontaneous tumors bearing dogs.

## MATERIALS AND METHODS

### Plasmids

pDUV5 encodes a modified canine TERT (cTERT) nucleotide sequence based on the isogenic human TERT construct [7]. Forty-seven amino acids were deleted in the N-ter region that encodes the nucleolar localization signal (NoLS), while three amino acids (VDD) were removed from the catalytic site (Figure 2A). The human ubiquitin (Ubi, 76 aa) was added to the N-terminus, the human and dog ubiquitin sequences being identical. An influenza (Flu) epitope restricted to HLA-A^*^0201 and a V5 tag was added at the C-terminal part of this fusion protein to facilitate biological and immunological characterization. The modified cTERT sequence was synthetized by GeneCust (Luxembourg) and subcloned into the expression vector pcDNA3.1(+) (Invitrogen, Carlsbad, USA) via the BamHI and XbaI sites. pNTC-DUV5 resulted from subcloning the pDUV5 insert into the expression vector NTC8685-eRNA41H-BamH1-Xbal vector (Nature Technology Corporation, Nebraska). This is a vector of choice for the induction of cellular immunity and as it does not encode and antibiotic resistant gene is environmentally safe.

### Cell culture

HEK293T (Human embryonic kidney) cell line and CRFK (Crandell-Rees feline kidney) cell line (from the ATCC) were cultured in Dulbecco’s Modified Eagle Medium (DMEM) supplemented with 10% heat-inactivated fetal calf serum (FCS), 1% sodium pyruvate, 1% penicillin/streptomycin pyruvate and 0.1% β-mercaptoethanol. QT6 cells (Japanese quail fibrosarcoma cell line) were cultured in HAM’s F10 medium (Eurobio, Courtaboeuf, France) supplemented with 10% FCS, 1% penicillin/streptomycin, 1% chicken serum, 1% L-glutamine and 0.5% tryptose broth. All components of the culture medium were purchased from Life technologies SAS (Saint-Aubin, France).

### Western blotting

To assess protein expression, HEK293T cells were transfected with pDUV5 using JetPRIME^®^ transfection reagent (Polyplus-transfection Inc., France). Cells were harvested from 24 hours post-transfection, lysed in a specific RIPA buffer (Sigma-Aldrich, St. Louis, USA) and expression assessed by western blotting assay. pDUV5 proteins were detected using a primary mouse anti-V5 antibody (R960-25; Invitrogen, Carlsbad, USA) followed by a secondary sheep anti-mouse antibody-horseradish peroxidase (HRP) conjugate (NA931; GE Healthcare, Buckinghamshire, UK). β-actin protein was used as loading control. Peroxidase activity was detected using a chemiluminescence ECL HRP substrate reagent kit (GE Healthcare, Buckinghamshire, UK).

### Immunofluorescence

For sub-cellular localization QT6 cells were transfected for 24 hours using Fugene HD transfection reagent (Promega, Charbonnières-les-Bains, France). After fixation, permeabilization and blocking steps, cells were incubated with a primary mouse anti-V5 antibody (R960-25; Invitrogen, Carlsbad, USA) or a rabbit anti-hTERT antibody (Y182, ab32020, Abcam, Cambridge, UK) for 1.5 hours followed by a goat anti-mouse antibody-Alexa Fluor 488 conjugate (A11029, Life Technologies, Saint-Aubin, France) or a goat anti-rabbit-Alexa Fluor 488 conjugate (A11008, Life Technologies, Saint-Aubin, France) for 45 minutes at room temperature. After washes, samples were mounted in DAPI-containing mounting medium (VECTASHIELD). Slides were analyzed by fluorescent microscopy (Axio observer Z1 and Axiovision, Carl Zeiss MicroImaging GmbH).

### TRAP assay

Telomerase activity was assessed on total cell protein extracts from CRFK cells (telomerase negative cells [40], transfected 24 h with pDUV5 or a wild type human TERT plasmid as a positive control, using the TeloTAGGG Telomerase PCR ELISAPLUS kit according manufacturer’s instructions (Roche Diagnostic GmbH, Mannheim, Germany). Briefly, protein extracts were used to evaluate the telomerase-mediated elongation of telomeric sequences. Products were amplified by PCR (30 cycles) using biotinylated primers. PCR amplification products were transferred to streptavidin pre-coated microplate, incubated with an anti-digoxigenin HRP linked antibody and revealed using TMB substrate. Absorbance was measured against a blank at 450 nm to determine the level of telomerase activity in each sample. Inactivated samples and lysis buffer served as negative controls. The relative telomerase activity (RTA) was obtained using the following formula:

RTA= [(AS-AS0)]/AS,IS]/[(ATS8-ATS8,0)/ATS8,IS] × 100

where AS: sample absorbance; AS0: heat-treated sample absorbance; AS,IS: internal standard sample absorbance; ATS8: control template absorbance; ATS8,0: lysis buffer (TS8) absorbance; ATS8,IS: TS8 IS absorbance.

### *In vivo* mouse studies

Six week old female C57BL/6JRj mice were purchased from Janvier laboratories (Saint-Berthevin, France). All experiments were conducted in strict accordance with the ethical guidelines and good animal practices of the European Committee (Directive 2010/63/EU). Mice were immunized twice at D0 and D14 by intradermal (ID) injection with 100 μg in 2 × 25 μL (bilateral injection) of pDUV5 or PBS as control, at the base of the tail. Immediately after ID vaccination, EGT was performed using Agilepulse*^®^ in vivo* system electroporator (BTX, USA). Invasive needle electrodes (6X4X2, 47-0050, BTX, USA) are inserted into the skin so that the injection site is placed between the two needle rows (the two needle rows are 0.4 cm apart). Two pulses of different voltages were applied: high voltage (HV) = 1125 V/cm (2 pulses, 50 μs-0.2 μs pulse interval) and low voltage (LV) = 250V/cm (8 pulses, 100 V-10 ms-20 ms pulse interval).

### *In vivo* dog studies

Six naïve beagle healthy dogs (3 males and 3 females; 24-34 months at the time of the first administration) were immunized ID 4 times at days 1, 29, 57 and 148 with 400 μg of pNTC-DUV5 as part of a sub-contract to the Centre de Recherches Biologique (CERB, Baugy, France). On each day of vaccination, 400 μg in 2 × 100 μl (bilateral injection) of pNTC-DUV5 was injected in each flank near the superficial inguinal lymph node in anaesthetized animal (DORBENE^®^, 20-80 μg/kg IV followed by IMALGENE^®^, 2.5 mg/kg, IV). Immediately after ID vaccination, EGT was performed using CLINIPORATOR^®^ 2 (IGEA, Carpi, Italy); one HV pulse (100 μs duration; 1,250 V/cm) followed 1,000 ms later by one LV pulse (400 ms duration; 180 V/cm) were applied with non-invasive plate electrodes (P-30-8G). At the end of the vaccination procedure, ALZANE® (100–400 μg/kg, IM), was administered to each animal to reverse the sedative and analgesic properties of medetomidine chloride. This study was approved by the CERB Internal Ethics Committee. Animal use and care are in accordance with the Directive 6/609/EEC European Convention for the Protection of Vertebrate Animals used for Experimental and Other Scientific Purposes. Blood was collected at specific time points and peripheral mononuclear cells (PBMC) were isolated using Ficoll. Frozen PBMC samples were analyzed for immunologic assays.

### Synthetic cTERT peptides

H2-Db restricted pDUV5 peptides used in mouse studies were determined *in silico* using four online algorithms (Syfpeithi, http://www.syfpeithi.de/; Bimas, http://www-bimas.cit.nih.gov/; NetMHCpan and SMM, http://tools.immuneepitope.org/main/). Synthetic lyophilized (>90% purity) peptides were purchases from Proimmune (Oxford, UK). The cTERT peptide library (70% purity) used in dog studies was purchased from GenScript (Piscataway, USA). Nineteen pools of peptides spanning the entire cTERT protein were used. Each pool was composed of 14 peptides of 15-mers overlapping by 11 amino acids. For *in vitro* immunization one pool of 18 peptides were used. All peptides were dissolved in sterile water at 2 mg/mL and stored at −80° C until use.

### *In vitro* immunization assay in dogs PBMCs

On day 0, dog frozen PBMCs were plated at 10^6^ cells/mL in 48-well flat bottomed plates in AIM-V medium (Invitrogen) supplemented with 100 ng/mL canine GM-CSF and 5 ng/mL canine IL-4 (R&D Systems) and cultured at 37° C, 5% CO_2_. After 24 hours, maturation stimuli were added, comprising the following reagent: 50 ng/mL canine TNFα, 20 ng/mL canine IL1-β (R&D Systems), 1 ng/mL human IL-7 (Miltenyi). Pool A peptides were also added at a final concentration of 10 μg/mL. Control wells received the cocktails of maturation cytokines only and no peptide. At day 3, culture medium was discarded and fresh AIM-V was added. Fresh AIM-V was added every 3 days until the day of testing. At day 15 after the beginning of culture, cells were recovered, washed in fresh AIM-V medium and used for the ELISpot assay.

### IFN-γ ELISpot assay

Blood sampling was performed on non-anesthetized animals from a peripheral vessel (saphenous, femoral or cephalic vein) at pre-dose and then at days 15, 22, 43 and 56. Hematology, coagulation parameters and blood chemistry analysis were performed. Murine IFN-γ and dog IFN-γ were purchased from Diaclone (Eurobio, Courtaboeuf, France) and R&D systems (Bio-Techne, Lille, France) respectively. They were used with Ficoll-purified lymphocytes from mouse spleen or dog PBMCs following the manufacturer’s instructions. Briefly, cells were stimulated in triplicate (mouse lymphocytes) or duplicate (dog PBMCs) at 2 × 10^5^ cells/well with individual cTERT peptide (mice) or pools of restricted cTERT peptides at 5 μg/mL. Serum free medium and phorbol 12-myristate 13-acetate (PMA)-ionomycin (50 and 500 ng/mL) were used as negative and positive controls respectively. Spots were counted using an automated ELISpot reader.

### *In vivo* cytotoxicity assay in pDUV5 immunized mice

The capacity of CD8 cytotoxic T-cells to kill peptide loaded target cells *in vivo* was assessed as described previously [41]. Briefly, splenocytes from naive C57BL/6 mice were split into three equal parts and each part was stained with carboxyfluorescein diacetate succinimidyl ester (CFSE) at 5 μM (high concentration), 1 μM (medium) or 0.2 μM (low). Subsequently, CFSE high-labeled cells were pulsed with the cTERT p621 peptide and CFSE medium-labeled cells were pulsed with p987 cTERT peptide for 1.5 hours whereas CFSE low-labeled cells were left unpulsed. Cells were mixed in a 1:1:1 ratio and 10^7^ cells were i.v. injected in 50 μL of PBS into control or pDUV5 immunized mice 10 days after the second immunization. Fifteen hours later, single cell suspensions from spleens were analyzed by MACSQUANT^®^ flow cytometer (Miltenyi, Germany). The percentage of specific killing was determined as follows:

[1 − [mean (%CFSE^low^/CFSE^high or medium^)control/(%CFSE^low^/CFSE^high or medium^)immunized]] × 100.

### Statistical analyses

Statistical analyses were performed by a two-tailed Mann Whitney non-parametric test using GraphPad prism 6.0 software (GraphPad Software Inc, USA). *p*-values ≤0.05 were considered significant.
